# Enhancing physicochemical properties of papaya through osmotic dehydration with various natural sweeteners

**DOI:** 10.1038/s41598-024-74605-z

**Published:** 2024-10-11

**Authors:** Damanpreet Kaur, Manpreet Singh, Ruchika Zalpouri, Preetinder Kaur, Raminder Singh Gill

**Affiliations:** 1 Department of Processing and Food Engineering, Punjab Agricultural University, Ludhiana, Punjab India; 2 Department of Renewable Energy Engineering, Punjab Agricultural University, Ludhiana, Punjab India; 3 Department of Mechanical Engineering, Punjab Agricultural University, Ludhiana, Punjab India

**Keywords:** Osmosis, Papaya, Jaggery, Honey, Optimization, Quality, Solar drying, Chemistry, Engineering

## Abstract

Osmotic processes play a crucial role in developing high-quality intermediate moisture food products. This study investigates the role of osmotic dehydration focusing on using natural sweeteners to reduce health risks from refined sugar. Jaggery and honey were used for osmosis of papaya cubes, with a Box-Behnken design to determine optimal conditions: osmosis temperature (30, 40, 50 °C), osmotic solution concentration (40, 50, 60°Brix), and osmosis time (3, 4, 5 h). Simultaneous optimization of these parameters considered responses such as water loss, solid gain, weight reduction, colour change, ascorbic acid content, lycopene content, and phenolic content. The optimized conditions were identified as 49.46 °C, 40°Brix, and 5 h for jaggery osmosed samples and 39.64 °C, 60°Brix, and 4.92 h for honey osmosed samples. Drying the osmosed samples using advanced domestic solar dryer revealed superior quality (total phenolic content and lycopene content) in jaggery osmosed papaya compared to honey osmosed papaya. The study suggests that introducing a new osmotic agent, jaggery, can enhance the nutritional value of osmosed papaya cubes.

## Introduction

Papaya (*Carica papaya* L.) is an important fruit in tropical and subtropical regions throughout the world with 138.4 thousand hectares of cultivation in India (Anonymous 2018). Papaya is rich in carbohydrates, vitamin A, calcium, iron, and fibre, as well as phosphorus, lipids, magnesium, and amino acids. Only papaya contains papain, a digestive enzyme that breaks down proteins and cleans the digestive tract^1^. Due to increased post-harvest metabolic activity, papaya is highly perishable resulting in low prices during peak season and huge financial losses due to spoilage. These fruits can be preserved in different ways, thereby reducing their wastage on one hand and enhancing their availability during off-season on the other hand.

The primary factor contributing to the perishability of fruits and vegetables is their elevated water content. Various methods, either individually or in combination, have been explored to extend the shelf life of fruits. Osmosis emerges as a preservation technique aimed at enhancing the longevity of fruits and vegetables by reducing their moisture content^2^. This process involves the partial removal of moisture through immersing plant tissues in a concentrated solution, a pre-treatment method long employed in the food industry^3^. Osmosis, being a neutral and non-destructive process, is utilized for extracting water from whole or cut fruits and vegetables^4^. The hypertonic solution’s higher osmotic pressure serves as a driving force, facilitating the simultaneous diffusion of water from the tissue into the solution and solutes from the osmotic solution into the tissue. However, the semi-selective nature of the osmotic membrane allows other solutes within the cells to leach into the osmotic solution^5,6^.

Various osmotic agents can be employed for osmosis, with the ideal characteristics being non-toxic, non-irritating, and possessing a pleasant taste and odor. Both honey and sucrose have been recognized as effective osmotic agents^7^. While sucrose, constituting 99% of refined sugar, has been traditionally used, it is deemed poor in nutrition, contributing to calorie intake associated with weight gain, diabetes, heart diseases, and dental problems^5^. Modern nutritional trends and a preference for a healthier lifestyle have led consumers to increasingly opt for sugar substitutes like honey and jaggery, which offer nutritional benefits^8^. Research indicates that jaggery is more nutrient-dense than refined sugar^9^. Honey, in addition to preventing enzymatic browning, enhances the organoleptic qualities of foods and does not crystallize on fruit surfaces^10^. These natural sweeteners, prized for their favourable sensory attributes and technological advantages as thickeners or humectants, are widely adopted by the food industry. They exhibit resistance to heat and pH changes and do not participate in the Maillard reaction, making jaggery and honey preferable sweetening agents over sucrose, both for direct consumption and in processed products such as sweets and confectionery^11^. Jaggery and honey are superior to refined sugar not only in terms of their nutrient density but also due to their additional health benefits, functional properties, and positive environmental and socio-economic impacts. These natural sweeteners contribute more than just sweetness to our diet; they offer essential nutrients, antioxidants, and health-promoting properties, making them a better choice for overall well-being^12,13^.

A technique for drying products using indirect solar radiation is efficient. This type of dryer uses flat plate collectors or concentrated solar collectors to heat the surrounding air. Hot air is then circulated in the cabinet where trays carry the produce to be dried^14^. Convection and diffusion are responsible for removing moisture from the product. It is done in an enclosed chamber so that there is no direct exposure of the product to solar radiation as a consequence, the disadvantages of direct solar drying method are overcome^15^.

In light of their positive impact on health, a research project was undertaken to enhance the efficiency of natural sweeteners in influencing the osmosis process of papaya fruit and various physical and biochemical attributes. These attributes encompassed total colour difference, solid gain, water loss, weight reduction, levels of ascorbic acid, lycopene, and total phenolic content. Additionally, the study involved monitoring the rate of pH, total soluble solids, and electrical conductivity variation under optimal osmotic conditions. Subsequently, the samples were subjected to solar drying to identify the most suitable intermittent moisture levels.

## Materials and methods

### Sample preparation and solutions for osmotic process

Fresh papaya (cv. Red Lady 786) was obtained from the Punjab Agricultural University farms in Ludhiana, India. The papayas underwent a thorough washing with water, followed by peeling using a hand peeler and removal of seeds. Subsequently, the papayas were precisely cut into cubes of nearly uniform size measuring 2 cm × 2 cm × 2 cm. These papaya cubes were then subjected to immersion in osmotic solutions of varying concentrations (40, 50, and 60°Brix), prepared using different osmotic agents, namely jaggery and honey. The ratio of the osmotic solution to the sample weight was maintained at 4:1 (v/w).

### Experimental procedure

The papaya cubes were dipped in osmotic solutions at three different temperatures (30, 40 and 50 °C) and for three different time intervals (3, 4 and 5 h). The papaya was kept completely submerged in the osmotic solutions to ensure uniform moisture removal. The samples were taken out of the solution, properly drained and surface dried, once the specified amount of osmosis had occurred.

### Experimental design

In present study, effect of three independent variables was evaluated viz. OT, Osc and Ot on colour change, solid gain, water loss, weight reduction, ascorbic acid content, lycopene content and total phenolic content of papaya using RSM. The experiments were performed in triplicate. Three level Box-Behnken design was used to generate seventeen experimental runs.

### Physical quality parameters

#### Total colour difference

Colour comparison was obtained by measuring L*, a*, and b* of the osmosed papaya samples using a colourimeter (CR-10 colour reader, Konica Minolta, Japan)^43^. The difference in colour of different papaya samples with respect to fresh papaya was calculated using Eq. (1).

1 $$\:\text{E}\:={\:\sqrt{{{{(L}_{2}^{*}-{L}_{1}^{*})}^{2}}\:+\:{({a}_{2}^{*}-{a}_{1}^{*})}^{2}\:+\:{({b}_{2}^{*}-{b}_{1}^{*})}^{2}}}$$

#### Solute gain, water loss and weight reduction

The kinetics of osmotic dehydration were effectively tracked through individual analyses conducted on each sample. Utilizing Eq. (2) to (4), data pertaining to water loss, solid gain, and weight reduction were gathered at different time points during the operations, specifically at 3, 4, and 5 h^16^.

2 $$\text{Water loss \%} = \frac{({\text{M}}_{o}-{\text{m}}_{o})-(\text{M}-\text{m})}{{\text{M}}_{o}}\times100$$

3 $$\text{Solid Gain (\%)}=\frac{({\text{m}}-{\text{m}}_{o})}{{\text{M}}_{o}}\times100$$

4 $$\text{Weight reduction (\%)}=\text{Water loss}-\text{Solid gain}$$

### Biochemical quality parameters

#### Ascorbic acid content

The determination of ascorbic acid content in papaya cubes was conducted following the method outlined by Ranganna^17^. 1 g of the sample was crushed in a pestle and mortar with 20 ml of acetic acid-metaphosphoric acid solution (450 ml of distilled water and 40 ml of acid in 5 g of acid), then the mixture was filtered. The filtered extract was titrated against the dye in 5 ml (52 mg of 2,6 dichloro-indophenol and 2 mg of sodium bicarbonate were dissolved in 200 ml of distilled water). Continue titrating until pink colour appears. In the sample, the amount of dye used to oxidize the vitamin C was noted. Ascorbic acid (0.2 mg/ml) standard volume was calculated using dye and titrating the standard solution. The ascorbic acid content of the osmosed sample was calculated using the following formula:

5 $$\:\text{A}\text{s}\text{c}\text{o}\text{r}\text{b}\text{i}\text{c}\:\text{a}\text{c}\text{i}\text{d}\:\text{c}\text{o}\text{n}\text{t}\text{e}\text{n}\text{t}\:(\text{m}\text{g}/100\text{g})\:=\:\frac{{\text{V}}_{2}\times\:\text{V}\times\:100}{{\text{V}}_{s}\times\:{\text{V}}_{1}\times\:\text{W}}$$

#### Lycopene content

The pigments were determined by using a method described by Nagata and Yamashita (1992). 1 g of fresh weight was homogenized separately with 10 ml of an acetone-hexane mixture (2:3) for 2 min. The absorbance spectra of the supernatant were measured using a UV-Visible spectrophotometer (Rayleigh UV-2601) and the absorption maxima were read at 453, 505, 645 and 663 nm. The following equations were used to calculate the amount of lycopene in the osmosed samples:

6 $$\:\text{L}\text{y}\text{c}\text{o}\text{p}\text{e}\text{n}\text{e}\:(\text{m}\text{g}/100\text{m}\text{l})=-0.0485\:{A}_{663}+0.204\:{A}_{645}+0.372\:{A}_{505}\:-0.0806\:{A}_{453}$$

#### Total phenolic content

The total phenolic content was measured with Folin-Ciocalteu reagent method McDonald et al.^19^. 1 g sample was crushed and mixed with 10 ml of methanolic water, prepared by mixing methanol and water in equal proportions. After extracts were filtered, 0.5 ml of the extract was mixed with 5 ml of diluted Folin-Ciocalteu reagent (1:10) and 4 ml of aqueous sodium carbonate (1 M). UV-Visible spectrophotometer was used to test the mixture’s absorbance at 765 nm in comparison to a blank after it had stood for 15 min. By using the gallic acid standard curve as a reference, the values were expressed as mg of gallic acid equivalent (GAE). The results were expressed as milligrams per 100 g.

7 $$\:\text{T}\text{o}\text{t}\text{a}\text{l}\:\text{p}\text{h}\text{e}\text{n}\text{o}\text{l}\text{i}\text{c}\:\text{c}\text{o}\text{n}\text{t}\text{e}\text{n}\text{t}\:(\text{m}\text{g}/100\text{g})\:=\:\frac{\text{C}\times\:\:\text{V}\:\times\:100}{\text{M}}$$

### Kinetics of optimized samples

Electrical conductivity serves as a metric for gauging the ease with which electric charge or heat can traverse a material, a characteristic influenced by factors such as the solution’s source, acidity, moisture, and viscosity. This parameter is integral to both quality control and quality assurance in the production process. The electrical conductivity of the osmotic solution was assessed using an electrical conductivity meter (FiveEasy Plus, Mettler Toledo, Mumbai, India).

The pH of a solution, denoting hydrogen ion concentration and thus its acidity, was determined using a digital pH meter (Type 101, Electronic Corporation of India Limited, Hyderabad, India). Total soluble solids (TSS) represent the entirety of soluble substances in a given volume of solution. In cases where sugar is the primary component, such as in sugar solutions like honey, juices, and syrups, the sugar content is measured. The index of refraction, indicative of the total soluble solids content, was employed to assess TSS in the osmotic solution. This was accomplished using a digital refractometer with a range of 0–93°Brix (Pocket refractometer PAL-3, Atago Co. Ltd., Japan), providing direct readings in °Brix. TSS measurement involved placing two or three drops of the solution on the fixed prism of the digital refractometer.

The rate of change of electrical conductivity, total soluble solids, and pH concerning time was monitored at 30-min intervals until the completion of the osmosis process.

### Optimization, validation procedure and statistical analysis

The SOP equation was fitted to the experimental data of each dependent variable:

8 $$\:{\text{Y}}_{\text{k}}={{\upbeta\:}}_{\text{o}}\:+\:\sum\:_{i=1}^{n}{\beta\:}_{i}{x}_{i}+\:\sum\:_{i=1}^{n}{\beta\:}_{ii}{x}_{i}^{2}+\:\sum\:_{i=1}^{n-1}\sum\:_{j=i+1}^{n}{\beta\:}_{ij}{x}_{i}{x}_{j}$$

The optimization of the osmosis process aimed to identify the optimal levels of independent variables (OT, Osc, and Ot) that would minimize y1 and y2 while maximizing y3, y4, y5, y6, and y7. To facilitate experimental design and factor optimization in relation to responses, Response Surface Methodology (RSM) was employed. The experimental data was analyzed using a commercial statistical tool, specifically Design-Expert version 7.0.0 by Stat-Ease Inc., Minneapolis, USA^20^. In the development of response surface and contour plots, RSM provided valuable insights for optimizing the factors. To ensure the accuracy of the model, all quality parameters were validated, and the model’s predictive capability was assessed by maintaining a percentage error between predicted and experimental values of less than 10%. This validation process adds robustness and reliability to the optimization outcomes^4^.

### Drying of sample using solar dryer

Following optimal osmosis treatment, the papayas were dried using the Advanced Domestic Solar Dryer (ADSD), an innovative device developed by the Department of Renewable Energy Engineering at Punjab Agricultural University, Ludhiana, India^21,44^. The ADSD boasts an aperture area of 0.40 m² and requires an unobstructed space measuring 80 cm by 66 cm. Its inclined absorber plate enhances the effective aperture area for solar energy input, surpassing solar dryers with a horizontal aperture. Inside the ADSD, the average temperature reaches 50 ± 5 °C. The drying process takes place within a cabinet, minimizing the risk of discolouration and effectively preserving nutrient content. The osmosed samples are placed on perforated trays in a separate drying chamber, ensuring they are not directly exposed to solar radiation. Heated air from the collector region passes through the crop during drying. The samples required two days to achieve the desired intermittent moisture content of 15–16% (wb). The solar radiation intensity varied from 110 to 740 W/m^2^ during day 1 and 110 to 720 W/m^2^ during day 2. To maintain reliability and consistency, the dried samples underwent triplicate analyses for total colour difference, ascorbic acid content, lycopene content, and total phenolic content.

## Results and discussion

The average values for the physical parameters of fresh papaya were determined as follows: moisture content at 87.17% (wb), L value at 33.00, a value at 11.80, and b value at 18.80. On the biochemical front, the parameters for fresh papaya were recorded as total soluble solids at 13.5°Brix, ascorbic acid content at 250 mg/100 g, lycopene content at 1.65 mg/100 g, and total phenolic content at 54 mg/100 g.

### Fitting the response surface model to significant independent variables

**Fig. 1:**
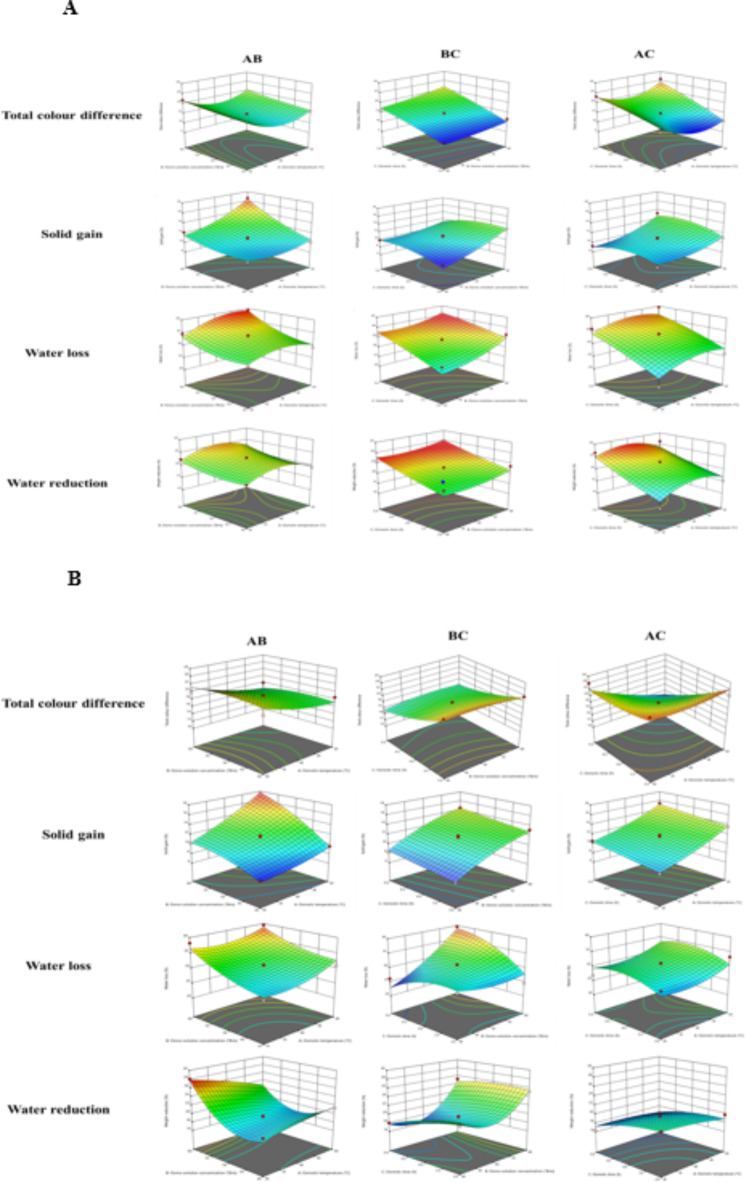
(**a**) The effect of OT, Osc and Ot on physical quality parameters of jaggery osmosed papaya. (**b**) The effect of OT, Osc and Ot on physical quality parameters of honey osmosed papaya.

**Fig. 2:**
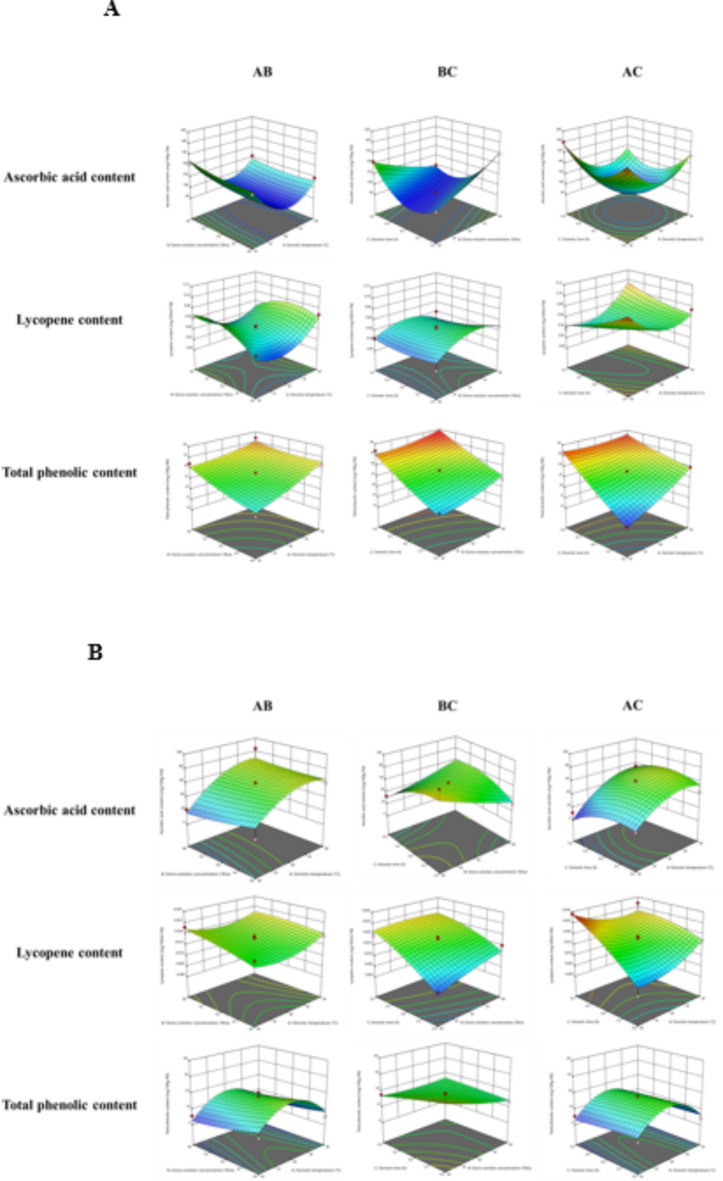
(**a**) The effect of OT, Osc and Ot on biochemical quality parameters of jaggery osmosed papaya. (**b**) The effect of OT, Osc and Ot on biochemical quality parameter of honey osmosed papaya.

The results indicate that the response surface models were statistically significant (*p* < 0.05) for all responses, demonstrating a high coefficient of determination (R^2^ > 0.992). The contour plots, depicted in Figs. 1 and 2, illustrate the impact of OT, Osc, and Ot on the physico-chemical parameters of jaggery and honey osmosed papaya samples.

### Effect of unconventional natural sweeteners on physical quality parameter of osmosed papaya

#### Total colour difference

Colour is a crucial visual indicator for evaluating the effects of heat treatment and predicting quality degradation from heat exposure. Tables 1 and 2 show that the total colour difference ranged from 9.1 to 25.9 for jaggery osmosed papaya and 11.7–24.3 for honey osmosed papaya. In jaggery osmosed papaya, the factors Osc, Ot, and the quadratic term OT significantly (*p* < 0.05) increased the total colour difference, while the quadratic term of Ot and all interaction terms significantly decreased it. The high F value for the main term of jaggery solution temperature indicates its significant influence on the total colour difference (Table 3). For honey osmosed papaya, the main terms OT and Ot, along with the interaction term of Ot and OT, significantly (*p* < 0.05) increased the total colour difference. Conversely, the factor Osc and all quadratic terms significantly decreased it. The high F value for Osc emphasizes its key role in influencing the total colour difference of honey osmosed papaya (Table 4). In the jaggery osmosed samples, the total colour difference increases with the rise in the concentration of the solution (Fig. 1a). Conversely, in honey osmo-solution, while there is a significant colour change with the increase in OT and time during the osmosis of papaya, the colour change diminishes with the increase in the solution concentration of honey (Fig. 1b). This phenomenon may be attributed to the fact that papaya tends to absorb the colour of the jaggery solution. Additionally, colour alteration can occur due to the micro-crystallization of the solute on the surface of the samples, as indicated by Tan et al.^22^. Comparable trends in colour change with an increase in osmo-solution concentration were also observed by Chauhan et al.^23^ in osmosed apple slices.

**Table 1:** The matrix of three-level box-behnken design and experimental data obtained for the response variables studied for jaggery osmosed papaya.

Y1	Y2	Y3	X1	X2	X3	X4	X5	X6	X7
30	40	4	20.2	10.00	29.43	19.43	132.21	0.036	18.66
30	50	3	14.3	9.02	21.02	11.99	178.29	0.106	13.46
30	50	5	23.2	9.28	35.90	26.62	180.29	0.037	36.67
30	60	4	21.8	12.11	34.43	22.32	144.23	0.061	32.08
40	40	3	9.1	8.23	29.22	20.99	96.15	0.011	18.66
40	40	5	15.8	9.81	35.63	25.82	144.23	0.025	36.86
40	50	4	14.6	10.63	33.58	22.95	84.13	0.041	27.75
40	50	4	14.4	11.02	33.93	22.91	84.11	0.044	27.81
40	50	4	14.6	10.71	33.72	23.01	84.14	0.047	27.71
40	50	4	14.7	10.64	33.59	22.95	84.10	0.045	27.75
40	50	4	14.4	10.79	33.36	22.57	84.09	0.042	27.79
40	60	3	11.6	12.83	36.40	23.57	158.65	0.048	23.66
40	60	5	20.9	10.39	38.05	27.66	98.56	0.040	38.42
50	40	4	16.3	10.01	29.08	19.07	117.79	0.068	31.49
50	50	3	10.7	10.96	26.08	15.12	153.85	0.074	29.69
50	50	5	25.9	13.25	39.59	26.34	127.40	0.088	37.53
50	60	4	19.1	16.62	39.26	22.64	122.60	0.043	36.94

**Table 2:** The matrix of three-level box-behnken design and experimental data obtained for the response variables studied for honey osmosed papaya.

Y1	Y2	Y3	X1	X2	X3	X4	X5	X6	X7
30	40	4	19.3	7.31	27.07	19.76	19.23	0.018	7.68
30	50	3	24.3	8.35	28.86	20.51	21.63	0.003	5.45
30	50	5	23.9	10.57	26.88	16.31	12.02	0.029	5.55
30	60	4	19.8	10.17	38.25	28.07	21.63	0.023	5.66
40	40	3	23.5	6.97	28.47	21.50	93.75	0.005	31.71
40	40	5	15.0	8.21	26.21	18.00	33.65	0.019	17.72
40	50	4	18.8	11.48	30.61	19.13	60.10	0.018	17.59
40	50	4	18.9	11.68	30.97	19.29	59.99	0.017	17.61
40	50	4	19.0	11.21	30.20	18.99	60.20	0.018	17.56
40	50	4	18.7	11.79	31.11	19.32	60.30	0.019	17.57
40	50	4	18.5	11.19	30.64	19.45	60.17	0.016	17.55
40	60	5	15.5	14.45	39.63	25.18	43.27	0.021	10.80
40	60	3	21.7	13.15	24.57	25.16	31.25	0.015	13.35
50	40	4	18.5	9.58	30.94	21.37	60.10	0.019	5.14
50	50	3	22.0	13.52	33.26	19.74	43.27	0.017	3.89
50	50	5	11.7	15.40	31.76	16.36	55.29	0.027	6.66
50	60	4	17.9	17.47	39.57	22.10	84.13	0.020	7.92

**Table 3:** ANOVA for quality parameters of jaggery osmosed papaya.

	Total Colour difference		Solid gain (%)		Water loss (%)		Weight reduction (%)		Ascorbic acid content (mg/100 g)		Lycopene content (mg/100 ml)		Total phenolic content (mg/100 g)	
	F value	*p* value	F value	*p* value	F value	*p* value	F value	*p* value	F value	*p* value	F value	*p* value	F value	*p* value
Model	18.53	0.0004*	13.07	0.0013*	7.17	0.0083*	33.32	< 0.0001*	4.22	0.0354*	4.55	0.0290*	55.07	< 0.0001*
A	3.61	0.0990	28.29	0.0011*	1.14	0.3192	54.42	0.0002*	2.64	0.1482	0.18	0.6807	44.76	0.0003*
B	103.34	< 0.0001*	50.25	0.0002*	2.85	0.1350	29.09	0.0010*	9.26	0.0188*	2.76	0.1405	3.80	0.0919
C	9.25	0.0188*	0.74	0.4173	2.53	0.1554	184.33	< 0.0001*	20.03	0.0029*	28.19	0.0011*	4.97	0.0609
AB	0.18	0.6800	10.53	0.0141*	5.27	0.0552	5.71	0.0481*	0.80	0.3982	0.02	0.8874	0.34	0.5729
AC	5.10	0.0584	2.14	0.1865	14.54	0.0066*	21.25	0.0025*	0.05	0.8187	0.54	0.4854	4.70	0.0667
BC	0.86	0.3823	8.40	0.0230*	1.02	0.3458	1.06	0.3365	0.68	0.4357	0.02	0.8775	78.69	< 0.0001*
A^2^	43.69	0.0003*	6.62	0.0368*	25.39	0.0015*	1.46	0.2651	2.88	0.1334	8.31	0.0235*	186.49	< 0.0001*
B^2^	0.21	0.6546	2.72	0.1430	12.20	0.0101*	1.65	0.2386	1.63	0.2413	1.20	0.3082	2.31	0.1723
C^2^	0.55	0.4796	8.76	0.0211*	1.16	0.3162	0.53	0.4902	0.18	0.6784	0.12	0.7383	144.89	< 0.0001*

A-Osmotic Temperature, B-Osmo-solution concentration, C-Osmosis time.

**Table 4:** ANOVA for quality parameters of honey osmosed papaya.

	Total colour difference		Solid gain (%)		Water loss (%)		Weight reduction (%)		Ascorbic acid content (mg/100 g)		Lycopene content (mg/100 ml)		Total phenolic content (mg/100 g)	
	F value	*p* value	F value	*p* value	F value	*p* value	F value	*p* value	F value	*p* value	F value	*p* value	F value	*p* value
Model	5.99	0.014*	104.64	< 0.0001*	4.22	0.0354*	27.37	0.0001*	4.28	0.0343*	6.88	0.0093*	4.41	0.0316*
A	13.12	0.008*	346.00	< 0.0001*	3.74	0.0943	5.26	0.0555	19.21	0.0032*	1.32	0.2880	0.00	0.9542
B	0.09	0.776	485.01	< 0.0001*	15.37	0.0057*	80.61	< 0.0001*	0.47	0.5131	4.28	0.0773	3.99	0.0859
C	28.60	0.001*	39.83	0.0004*	1.55	0.2529	24.95	0.0016*	1.41	0.2730	41.45	0.0004*	1.24	0.3022
AB	0.11	0.752	45.72	0.0003*	0.23	0.6444	23.44	0.0019*	0.63	0.4518	0.42	0.5362	0.31	0.5975
AC	8.69	0.021*	0.21	0.6615	0.01	0.9302	0.27	0.6166	0.63	0.4518	6.77	0.0354	0.09	0.7673
BC	0.47	0.515	0.01	0.9380	10.72	0.0136*	5.05	0.0594	7.06	0.0326*	1.69	0.2345	1.74	0.2290
A^2^	1.01	0.348	6.54	0.0376*	2.08	0.1920	0.71	0.4286	5.60	0.0499*	4.56	0.0701	32.14	0.0008*
B^2^	0.79	0.402	19.54	0.0031*	1.16	0.3163	105.01	< 0.0001*	0.07	0.7963	0.28	0.6100	0.23	0.6457
C^2^	1.13	0.322	0.02	0.8889	3.40	0.1077	3.23	0.1154	2.99	0.1272	1.44	0.2688	0.01	0.9285

A-Osmotic temperature, B-Osmo-solution concentration, C-Osmosis time.

$${E_{JOP}} = + 16.56 - 0.94{{~}}A + 1.50B + 5.01{{~}}C - 0.38AB - 3.48AC - 0.87BC + 0.81{A^2} - 0.70{B^2} - 0.85{C^2}$$

$$\:{E}_{HOP}=+18.78- 2.15A-\:0.18B+3.17C\:-0.28AB\:-2.48AC+0.57BC\:-0.82{A}^{2}\:-0.73{B}^{2}\:-0.87{C}^{2}$$

#### Solid gain

Tables 1 and 2 reveal that the solid gain ranged from 8.23 to 16.62% for jaggery osmosed papaya and 6.97–17.47% for honey osmosed papaya. In jaggery osmosed papaya, the main factors OT and Osc, along with interaction terms (i.e. OT and Ot; Osc, and Ot) and the quadratic terms OT and Ot, significantly (*p* < 0.05) increased solid gain. Conversely, the main term Ot, the interaction term OT and Ot, and the quadratic term Osc significantly decreased solid gain. The high F value for the main term Ot underscores its predominant influence on solid gain in jaggery osmosed papaya (Table 3). For honey osmosed papaya, all main factors and the interaction term Ot and Osc, as well as the quadratic terms OT and Osc, significantly (*p* < 0.05) increased solid gain. Conversely, the interaction term OT, Ot, and Osc, and the quadratic terms Ot, significantly decreased solid gain. The high F value for the quadratic term Ot indicates its dominant role in affecting solid gain in honey osmosed papaya (Table 4). In jaggery osmosed papaya, there is an observed increase in solid gain as Ot decreases (Fig. 1a). On the other hand, in honey osmosed papaya, the solid gain increases with the rise in osmosis time (Ot), osmotic solution temperature (OT), and osmosis concentration (Osc) (Fig. 1b). This phenomenon may be attributed to the collapse of the cell membrane at higher temperatures, influenced by the high concentration difference between solids in the fruit and the syrup, particularly at elevated syrup concentrations. This collapse of the cell membrane is more pronounced at high temperatures, as discussed by Kaleemullah et al.^24^. A similar trend was noted by Kaur et al.^25^ in kiwifruit.

$${\text{S}}{{\text{G}}_{{\text{JOP}}}}{\mkern 1mu} = {\mkern 1mu} + {\mkern 1mu} 10.76{\mkern 1mu} + {\mkern 1mu} 1.30{\text{A}}{\mkern 1mu} + {\mkern 1mu} 1.74{\text{B}}{\mkern 1mu} + {\mkern 1mu} 0.21~{\text{C}}{\mkern 1mu} + {\mkern 1mu} 1.13{\text{AB}}{\mkern 1mu} + {\mkern 1mu} 0.51{\text{AC}} - {\mkern 1mu} 1.00{\text{BC}}{\mkern 1mu} + {\mkern 1mu} 0.87{{\text{A}}^2}{\mkern 1mu} + {\mkern 1mu} 0.56{{\text{B}}^2} - {\mkern 1mu} 1.00{{\text{C}}^2}$$

$${\text{SG}}_{{\text{HOP}}} = + 11.47+ 2.45{\text{A}} + 2.90{\text{B}} +0.83{\text{C}} - 1.26{\text{AB}} - 0.09{\text{AC}} +0.02{\text{BC}}+0.46{{\text{A}}^{2}} - 0.80{{\text{B}}^{2}} + 0.03{{\text{C}}^{{2}}}$$

#### Water loss

In jaggery osmosed papaya, all quadratic terms and the interaction term OT and Ot significantly (*p* < 0.05) increased water loss. Tables 1 and 2 show that water loss ranged from 21.02 to 39.59% for jaggery osmosed papaya and 24.57–39.63% for honey osmosed papaya. Conversely, all main terms and the interaction terms (i.e., OT and Osc; Osc and Ot) significantly decreased water loss. The high F value for the quadratic term OT indicates its dominant influence on water loss in jaggery osmosed papaya (Table 3). For honey osmosed papaya, the main term Osc and the interaction term Ot, Osc significantly (*p* < 0.05) increased water loss. In contrast, the main term OT, the interaction terms OT, Ot, and Osc, and all quadratic terms significantly decreased water loss. The high F value for the interaction term Osc highlights its predominant role in influencing water loss in honey osmosed papaya (Table 4). In both jaggery and honey osmosed samples, water loss increased with the rise in Ot (Fig. 1a and b). Additionally, water loss during papaya osmosis increased with an increase in solution concentration. This can be attributed to the osmotic gradient that results from an increase in solution concentration, creating a higher driving force for water removal between the solution and the fruit, consequently leading to higher mass transfer rates. This trend aligns with findings reported by Shalini et al.^26^ in osmosed papaya, where water loss increased with an increase in Osc.

$${\text{W}}{{\text{L}}_{{\text{JOP}}}}\,=\,+\,{\text{33}}.0{\text{7}}\,+\,{\text{1}}.{\text{65A}}\,+\,{\text{3}}.10{\text{B}}\,+\,{\text{4}}.56{\text{C}}\,+\,{\text{2}}.85{\text{AB}} - \,{\text{3}}.21{\text{AC}}\,+\,0.53{\text{BC}}\,+\,{\text{1}}.39{{\text{A}}^{\text{2}}}\,+\,{\text{3}}.0{\text{2}}{{\text{B}}^{\text{2}}} - 0.{\text{6}}0{{\text{C}}^{\text{2}}}$$

$${\text{W}}{{\text{L}}_{{\text{HOP}}}}\,=\,+\,{\text{31}}.12\,+\,{\text{1}}.81{\text{A}}\,+\,{\text{3}}.67{\text{B}}\,+\,{\text{1}}.17{\text{C}}\,+\,{\text{2}}.55{\text{AB}} - \,{\text{2}}.21{\text{AC}}\,+\,0.43{\text{BC}}\,+\,{\text{1}}.29{{\text{A}}^{\text{2}}}\,+\,{\text{3}}.11{{\text{B}}^{\text{2}}} - \,0.{\text{6}}0{{\text{C}}^{\text{2}}}$$

#### Weight reduction

Tables 1 and 2 show that weight reduction ranged from 11.99 to 27.66% for jaggery osmosed papaya and 16.31–28.07% for honey osmosed papaya. In jaggery osmosed papaya, all main terms and the interaction terms OT, Osc, and OT, Ot exhibited a significantly (*p* < 0.05) positive effect on weight reduction. Conversely, the interaction term Osc, Ot, and all quadratic terms had a significantly negative effect on weight reduction. The higher F value for the interaction term Ot indicates its dominant influence on weight reduction in jaggery osmosed papaya (Table 3). In honey osmosed papaya, the main terms Osc and Ot, the quadratic term Osc, and the interaction term Osc, OT showed a significant (*p* < 0.05) positive effect on weight reduction (Table 4). However, the main term OT, the quadratic terms OT and Ot, and the interaction terms OT, Ot, and Osc, Ot had a significant negative effect. The high F value for the interaction term Osc suggests its substantial impact on weight reduction in honey osmosed papaya. Figure 1a showed that weight reduction in jaggery osmosed samples increases with an increase in Ot, whereas in Fig. 1b showed that honey osmosed papaya, it increases as the concentration and time of osmosis increase. This may be attributed to the fact that weight reduction increases with the concentration of sugar, aligning with basic theories that posit the concentration and temperature of the osmotic solution as determinants of the driving force for osmotic mass transfer. Increased temperature and solute concentration lead to a higher osmotic pressure gradient, thereby enhancing mass transfer. Shalini et al.^26^ also found a comparable trend in the weight reduction of papaya during osmosis, and Kumar et al.^27^ quoted the same trend for papaya.

$${\text{W}}{{\text{R}}_{{\text{JOP}}}}\,=\,+\,{\text{22}}.{\text{12}}\,+\,0.{\text{35A}}\,+\,{\text{1}}.{\text{36B}}\,+\,{\text{4}}.35{\text{C}} - \,{\text{1}}.89{\text{AB}}\,+\,0.{\text{2}}0{\text{AC}}\,+\,0.{\text{88BC}} - \,0.{\text{32}}{{\text{A}}^{\text{2}}}\,+\,{\text{3}}.{\text{91}}{{\text{B}}^{\text{2}}} - \,0.{\text{69}}{{\text{C}}^{\text{2}}}$$

$${\text{W}}{{\text{R}}_{{\text{HOP}}}}\,=\,+\,{\text{19}}.24-0.63{\text{A}}\,+\,{\text{2}}.49{\text{B}} - \,{\text{1}}.38{\text{C}} - \,{\text{1}}.49{\text{AB}}\,+\,0.{\text{4}}0{\text{AC}}\,+\,0.{\text{87BC}} - \,0.{\text{22}}{{\text{A}}^{\text{2}}}\,+\,{\text{3}}.71{{\text{B}}^{\text{2}}} - \,0.{\text{79}}{{\text{C}}^{\text{2}}}$$

### Effect of unconventional natural sweeteners on biochemical quality parameter of osmosed papaya

#### Ascorbic acid content (mg/100 g FW)

Tables 1 and 2 show that ascorbic acid content ranged from 84.09 to 180.29 mg/100 g FW for jaggery osmosed papaya and 12.02–93.75 mg/100 g FW for honey osmosed papaya. In jaggery osmosed papaya, the main terms Osc and Ot significantly (*p* < 0.05) increased ascorbic acid content, while the main term OT and all interaction and quadratic terms significantly decreased it. The higher F value for the quadratic term Osc highlights its dominant influence on ascorbic acid content in jaggery osmosed papaya (Table 3). For honey osmosed papaya, the main and quadratic terms OT, and the interaction term Osc, Ot, significantly increased ascorbic acid content (Table 4). Conversely, the main and quadratic terms Osc and Ot, and the interaction terms OT, Osc, and OT, Ot, significantly decreased it. The higher F value for the interaction term Osc indicates its substantial impact on ascorbic acid content in honey osmosed papaya. For both the samples, ascorbic acid content in papaya cubes decreased with the duration of osmosis due to leaching of organic acids in osmotic solution (Fig. 2a, b). However, in jaggery osmosed samples, a higher ascorbic acid content was observed at lower solution concentrations (Fig. 2a). This could be explained by the fact that the lesser concentration of the osmotic solution was unable to effectively coat the sample’s surface, leading to more ascorbic acid degradation. Similar results were reported by Islam et al.^28^ for osmotic dried papaya.

$${\text{A}}{{\text{A}}_{{\text{JOP}}}}\,=\,+\,{\text{84}}.11-{\text{14}}.42{\text{A}}\,+\,{\text{4}}.21{\text{B}} - \,{\text{4}}.81{\text{C}} - \,{\text{1}}.80{\text{AB}} - \,{\text{6}}.61{\text{AC}} - \,{\text{27}}.04{\text{BC}} - \,{\text{4}}0.58{{\text{A}}^{\text{2}}}\,+\,{\text{4}}.52{{\text{B}}^{\text{2}}}\,+\,{\text{35}}.{\text{77}}{{\text{C}}^{\text{2}}}$$

$${\text{A}}{{\text{A}}_{{\text{HOP}}}}\,=\,+\,{\text{48}}.23-{\text{21}}.04{\text{A}} - \,{\text{3}}.31{\text{B}} - \,{\text{5}}.71{\text{C}} - {\text{ 1}}.80{\text{AB}} - \,{\text{6}}.61{\text{AC}} - \,{\text{27}}.04{\text{BC}} - \,{\text{4}}0.58{{\text{A}}^{\text{2}}}\,+\,{\text{4}}.52{{\text{B}}^{\text{2}}}\,+\,{\text{35}}.77{{\text{C}}^{\text{2}}}$$

#### Lycopene content (mg/100 ml FW)

Tables 1 and 2 illustrate that lycopene content ranged from 0.011 to 0.106 mg/100 ml FW for jaggery osmosed papaya and 0.003–0.029 mg/100 ml FW for honey osmosed papaya. In jaggery osmosed papaya, the main term Ot and the quadratic term OT exhibited a significant (*p* < 0.05) positive effect on lycopene content. However, the main terms OT, Osc, and all interaction and quadratic terms involving Osc and Ot had a significant negative effect. The higher F value for the main term OT indicates its dominant influence on lycopene content in osmosed papaya (Table 3). For honey osmosed papaya samples, the main term Ot showed a significant (*p* < 0.05) positive effect on lycopene content. Conversely, the main terms OT, Osc, and all interaction and quadratic terms had a significant negative effect. The higher F value for the main term OT highlights its predominant role in affecting lycopene content in honey osmosed papaya (Table 4). The lycopene content of osmosed papaya increased with increasing Ot in honey osmotic solution, which contributed to overall solid gain and increased lycopene content in papaya samples (Fig. 2b). This is consistent with findings reported by Hempel et al.^29^ for peach palm fruit. However, in jaggery solution samples, the lycopene content of papaya samples decreased with an increase in osmo-solution concentration, Ot, and OT (Fig. 2a). This decrease in lycopene content is attributed to the increase in the temperature of the solution, causing a rise in the rate of cell breakdown and thermal degradation, ultimately leading to a decrease in lycopene content.

$${\text{L}}{{\text{C}}_{{\text{JOP}}}}\,=\,+\,0.0{\text{44}}-0.00{\text{4A}}\,+\,0.00{\text{6B}}-0.00{\text{6C}} - \,0.0{\text{1AB}}\,+\,0.0{\text{2AC}}-0.00{\text{5BC}} - \,0.0{\text{3}}{{\text{A}}^{\text{2}}} - \,0.0{\text{2}}{{\text{B}}^{\text{2}}}\,+\,0.00{\text{5}}{{\text{C}}^{\text{2}}}$$

$$\begin{aligned}{{\text{L}}{{\text{C}}_{{\text{HOP}}}}\,=\,+\,0.030\, - \,0.012{\text{ A}}\,+\,0.011{\text{B}}\,+\,0.014{\text{C}}\,+\,6.250{\text{E}}-003{\text{A}}{\text{B}}\,+\,7.000{\text{E}}-003{\text{A}}{\text{C}}} +\,4.500{\text{E}}-003{\text{B}}{\text{C}}\,  \\ - \,0.020{{\text{A}}^{\text{2}}}\,-\,6.925{\text{E}}-003{{\text{B}}^{\text{2}}}\,+\,8.250{\text{E}}-004{{\text{C}}^{\text{2}}}\end{aligned}$$

#### Total phenolic content (mg/100 g)

Tables 1 and 2 demonstrate that weight reduction varied from 13.46 to 38.42 mg/100 g FW for jaggery osmosed papaya and 3.89–31.71 mg/100 g FW for honey osmosed papaya. In jaggery osmosed papaya, the main term OT and the interaction term Osc, Ot, along with the quadratic term Ot, significantly (*p* < 0.05) enhanced the total phenolic content. However, the interaction terms OT, Osc, and OT, Ot had a significantly negative effect on the total phenolic content of jaggery osmosed papaya. The higher F value for the quadratic term Ot suggests its predominant influence on total phenolic content in osmosed papaya (Table 3). Regarding honey osmosed papaya samples (Table 4), all main, interaction, and quadratic terms exhibited a significant negative effect (*p* < 0.05) on total phenolic content. The higher F value for the interaction term Ot and OT indicates its prominent impact on total phenolic content in honey osmosed papaya. In jaggery osmosed samples, phenolic content increased with increasing solution concentration (Fig. 2a). Conversely, in honey osmosed papaya samples, phenolic content increased with decreasing Osc, Ot, and OT (Fig. 2b). This can be attributed to the fact that the higher concentration of the osmotic solution effectively coated the samples during the osmosis process, resulting in a reduction in the degradation of phenolic content^28^.

$${\text{TP}}{{\text{C}}_{{\text{JOP}}}}\,=\,+\,{\text{29}}.00\,+\,4.35{\text{A}}\,+\,3.18{\text{B}} - \,{\text{8}}.00{\text{C}} - \,0.10{\text{AB}}\,+\,0.05{\text{AC}}\,+\,0.19{\text{BC}} - \,0.26{{\text{A}}^{\text{2}}} - \,0.14{{\text{B}}^{\text{2}}}\,+\,0.09{{\text{C}}^{\text{2}}}$$

$${\text{TP}}{{\text{C}}_{{\text{HOP}}}}\,=\,+\,{\text{17}}.58-0.09{\text{A}} - \,3.07{\text{B}} - \,1.71{\text{C}}\,+\,1.20{\text{AB}}\,+\,0.67{\text{AC}}\,+\,2.86{\text{BC}} - \,{\text{11}}.99{{\text{A}}^{\text{2}}}\,+\,1.02{{\text{B}}^{\text{2}}} - \,0.20{{\text{C}}^{\text{2}}}$$

### Optimized condition

In the pursuit of achieving optimal product quality, the optimization strategy prioritizes the minimization of total colour difference and solid gain percentage, integral factors that significantly influence the visual appeal and textural characteristics of the end product. Simultaneously, the focus is on minimizing water loss percentage and maximizing weight reduction percentage to streamline processing efficiency and achieve an economically advantageous reduction in product weight. Additionally, the optimization objectives center around maximizing the concentrations of critical components, such as ascorbic acid, lycopene, and total phenolic content per unit weight, recognizing their pivotal roles in enhancing the nutritional profile and health-promoting attributes of the final product. The optimized operating conditions were found to be 49.46 °C, 40^o^Brix and 5 h for jaggery osmosed samples and 39.64 °C, 60°Brix and 4.92 h for honey osmosed samples (Table 5).

**Table 5:** Optimum values of process parameters.

Independent variable		Optimum values	Experimental values	Error percentage (%)	Optimum values	Experimental values	Error percentage (%)
		Jaggery osmotic solution			Honey osmotic solution		
OT (°C)	Is in range	49.46	50	1.09	39.64	40	0.91
Osc (°Brix)	Is in range	40	40	0.00	60	60	0.00
Ot (h)	Is in range	5	5	0.00	4.92	5	1.62
Responses							
Total colour difference	Minimize	19.96	19.5	2.36	16.61	16.5	0.67
Solid gain (%)	Minimize	10.78	10.4	3.65	14.05	13.89	1.15
Water loss (%)	Maximize	36.16	38.05	4.97	38.44	38.31	0.34
Weight reduction (%)	Maximize	25.37	25.66	1.13	24.99	25.16	0.68
Ascorbic acid content (mg/100 g FW)	Maximize	150.65	147.79	1.94	60.46	59.27	2.01
Lycopene content (mg/100 ml FW)	Maximize	0.06	0.021	0.07	0.02	0.021	4.76
Total phenolic content (mg/100 g FW)	Maximize	37.49	36.89	1.63	16.81	16.2	3.77

### Kinetics study during osmosis at optimium condition

#### pH

**Fig. 3:**
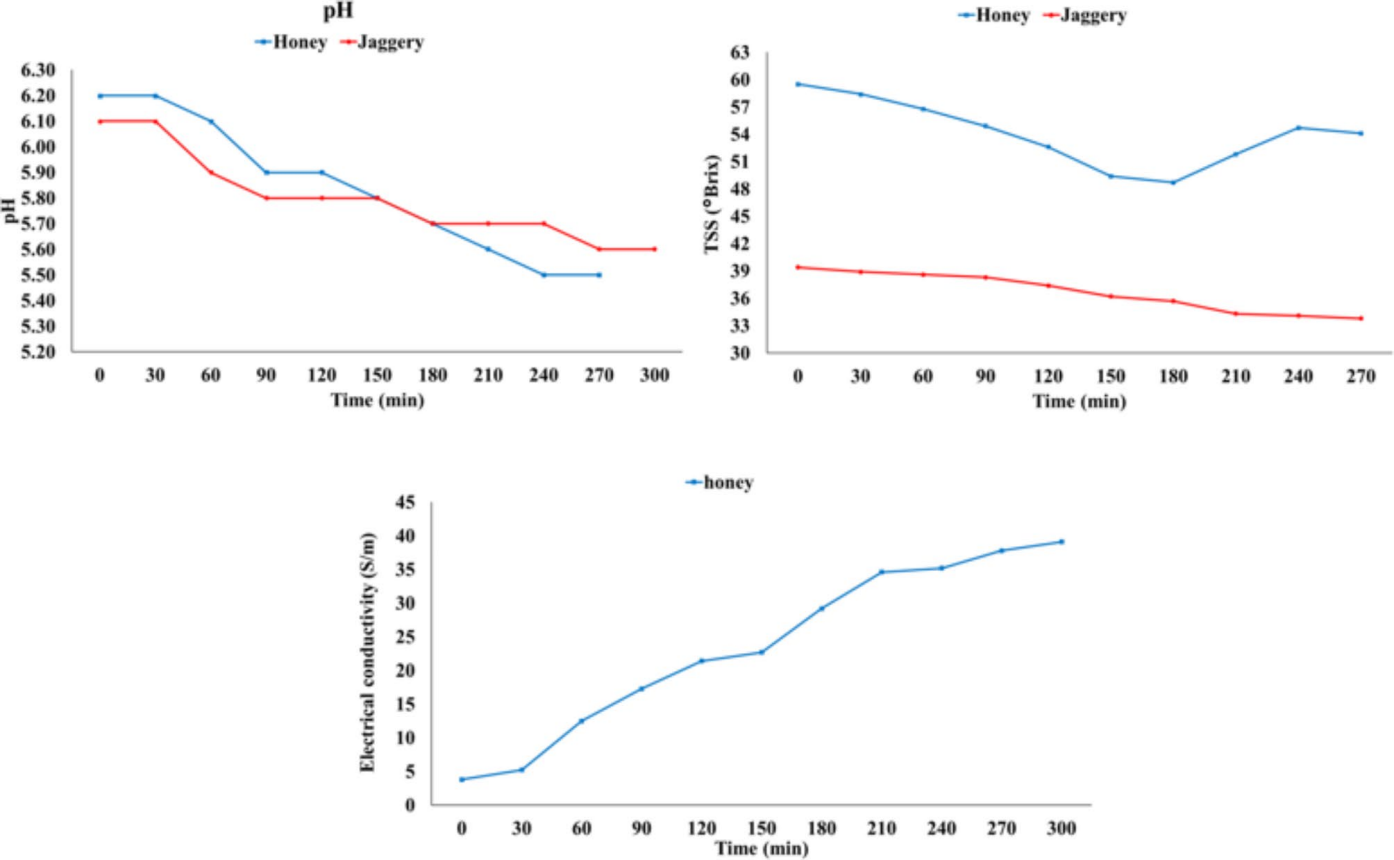
Trend for pH v/s time, total soluble solids v/s time and electrical conductivity v/s time.

The kinetic study investigated the relationship between pH and time, with pH measurements taken every 30 min. Figure 3 demonstrates that in both jaggery and honey solutions, the pH stabilized after 270 min. Overall, the pH of the osmotic solution decreased with time. This decline can be attributed to soluble acids from papaya leaching into the solution, causing the pH of the solution to shift from slightly acidic to moderately acidic^30^. The highest R^2^ values were obtained for the exponential model and polynomial (2nd order) model for honey (0.979) and jaggery (0.934) solutions, respectively. In contrast, the linear model had the lowest R^2^ value for both honey and jaggery solutions. Consequently, the exponential order was chosen for the kinetic study of pH in honey osmotic solution with respect to Os and the polynomial (2nd order) model for jaggery osmotic solution with respect to Os.

#### Total soluble solids (°Brix)

The kinetic study explored the relationship between Total Soluble Solids (TSS) and time, revealing a gradual decrease in TSS of osmotic solutions from 0 to 180 min (Fig. 3). This decline could be attributed to osmosis causing an increase in solid gain. After a certain time period, the crop acts as a higher concentration medium, while the solution acts as a lower concentration medium, leading to reverse osmosis and an increase in the concentration of the solution^31,32^. In contrast, Osc decreased with time and became constant after 240 min, and Osc decreased with time and became constant after 270 min. This phenomenon may be due to an increase in the movement of solids from the osmotic solution to the samples^31^. The highest R^2^ values were obtained for the polynomial (2nd order) model for both honey (0.816) and jaggery (0.973) solutions, respectively, while the lowest R^2^ values were found for the exponential and linear models for honey and jaggery solutions, respectively. Consequently, the polynomial (2nd order) model was chosen for the kinetic study of TSS in honey osmotic solution with respect to Os and the polynomial (2nd order) model for jaggery osmotic solution with respect to Os.

#### Electrical conductivity (S/m)

The kinetic study examined the relationship between electrical conductivity and time. Electrical conductivity (EC) of honey osmotic solution exhibited an increase with time (Fig. 3). This rise in EC can be attributed to the increase in the moisture content of the solution. During the osmosis process, there is a transfer of moisture from the fruit to the solution. As a result, the moisture content of the solution increases, leading to an increase in electrical conductivity Rao et al.^33^. The highest R^2^ value was obtained for the polynomial (2nd order) model for honey (0.988) solution, while the lowest R^2^ value was found for the exponential model for honey solution. Notably, there was no electrical conductivity observed in the jaggery solution.

### Drying of osmosed papaya at optimum condition using solar dryer

#### Total colour difference

Table 6 showed that the value of total colour difference was observed as 16.30 for jaggery osmosed papaya samples and 15.40 for honey osmosed papaya samples. It was observed that jaggery osmosed dried samples had maximum total colour difference from the fresh samples. This can be due to micro-crystallization of the solute on the surface of papaya samples that led to increase in the L value of solar dried samples^2^. Similar results were quoted by Chauhan et al.^23^ for apple, Kaur et al.^4^ for peas, Torres et al.^34^ for mango and Singh et al.^35^ for ber.

**Table 6:** Quality parameters of osmo-solar dried product.

Parameter	Jaggery osmosed solar dried sample	Honey osmosed solar dried sample
Total phenolic content (mg/100 g DW)	27.39 ± 0.25^a^	18.15 ± 0.15^b^
Total colour difference	16.30 ± 0.21^a^	15.40 ± 0.12^b^
Lycopene content (mg/100 ml)	0.028 ± 0.12^a^	0.008 ± 0.11^b^
Ascorbic acid content (mg/100 g DW)	48.09 ± 0.11^b^	72.15 ± 0.14^a^

Mean ± standard deviation; similar letters in column have non-significant effect.

#### Ascorbic acid content

The maximum value of ascorbic acid content was observed in honey osmosed papaya samples (72.15 mg/100 g DW) and minimum value was for jaggery osmosed papaya samples (48.09 mg/100 g DW) (Table 6). It can be attributed to the increase in osmotic temperature, thermal degradation increases resulting degradation in ascorbic acid content^28^. Similarly, it was shown by Islam et al.^28^ for papaya, Nuñez-Mancilla et al.^36^ for strawberry, Choudhary et al.^37^ for pineapple slices.

#### Lycopene content

Table 6 showed that the value of lycopene content was observed as 0.028 mg/100 ml for jaggery osmosed papaya samples and 0.008 mg/100 ml for honey osmosed papaya samples. The value of lycopene content was maximum for jaggery osmosed samples. During the solar drying process, due to enzymatic reactions, the decomposition of pigments occurred Seerangurayar et al.^38^. Similar results were quoted by Seerangurayar et al.^38^ for dates, Mendelová et al.^39^ for tomato.

#### Total phenolic content

Table 6 showed that the value of total phenolic content was observed as 27.39 mg/100 g DW for jaggery osmosed papaya samples and 18.15 mg/100 mg DW for honey osmosed papaya samples. It was observed that honey osmosed dried samples had minimum total phenolic content. The decrease in total phenolic content can be due to the transfer of water during the application of these treatments which have caused nutrient loss Bozkir et al.^40^. Similar results were quoted by Minuye et al.^41^ in papaya, Bozkir et al.^40^ for persimmon, Guerra-Valle et al.^42^ for apple.

## Conclusion

The Response Surface Methodology (RSM) demonstrated effective performance in optimizing the variables for converting papaya into a nutritionally enhanced product. This study highlighted that jaggery serves as an excellent alternative to non-nutritive osmotic agents. The optimized operating conditions were identified as 49.46 °C, 40°Brix, and 5 h for jaggery osmosed samples and 39.64 °C, 60°Brix, and 4.92 h for honey osmosed samples. The best model fitting for total soluble solids (TSS), pH, and electrical conductivity (EC) for honey was observed for the polynomial (2nd order), exponential, and linear models, respectively. Meanwhile, the best model fitting for TSS and pH for jaggery was observed for the polynomial (2nd order) model. The quality of jaggery osmosed dried product was found to be superior to honey samples, although the ascorbic acid content was higher in honey osmosed dried product. The information gathered in this study holds significance for the development of new food products and enhancing the value of existing ones. Furthermore, the exploration of jaggery as an osmotic agent in food product development remains an understudied area, warranting further research to maximize its health benefits across various fruits and processing methods.

## Data Availability

The datasets generated during and/or analyzed during the current study are available from the corresponding author on reasonable request.

## Abbreviations

JOP: Jaggery osmosed papaya
HOP: Honey osmosed papaya
A or OT or x_1_: Osmotic temperature
B or Osc or x_2_: Osmo-solution concentration
C or Ot or x_3_: Osmosis time
SOP: Second-order polynomial
RSM: Response surface methodology
Y_k_: Response variable
x: Independent variables
ΔE or y_1_: Total colour difference
SG or y_2_: Solid gain
WL or y_3_: Water loss
WR or y_4_: Weight reduction
AA or y_5_: Ascorbic acid content
LC or y_6_: Lycopene content
TPC or y_7_: Total phenolic content
M_o_: Initial mass of fresh fruit before the osmotic treatment
M: Mass of the fruit after time “t” of osmotic treatment
m: Dry mass of fruit after time “t” of osmotic treatment
m_o_: Dry mass of fresh fruit
V: Total volume of metaphosphoric acid (ml)
V_1_: Volume of extract (ml)
V_2_: Volume of dye used (ml)
V_s_: Volume of standard (ml)
W: Weight of sample taken (g)
FW: Fresh weight
DW: Dry weight
A_x_: (Absorbance)_wavelength_
C: Concentration of gallic acid from curve (mg/ml)
V: Volume of extract (ml)
M: Weight of sample total soluble solids extract (g)
ADSD: Advanced domestic Solar Dryer
